# An Updated Review on Marine Anticancer Compounds: The Use of Virtual Screening for the Discovery of Small-Molecule Cancer Drugs

**DOI:** 10.3390/molecules22071037

**Published:** 2017-06-23

**Authors:** Verónica Ruiz-Torres, Jose Antonio Encinar, María Herranz-López, Almudena Pérez-Sánchez, Vicente Galiano, Enrique Barrajón-Catalán, Vicente Micol

**Affiliations:** 1 Institute of Molecular and Cell Biology (IBMC), Miguel Hernández University (UMH), Avda. Universidad s/n, Elche 03202, Spain; vruiz@umh.es (V.R.-T.); jant.encinar@umh.es (J.A.E.); mherranz@umh.es (M.H.-L.); almudena.perez@umh.es (A.P.-S.); vmicol@umh.es (V.M.); 2 Physics and Computer Architecture Department, Miguel Hernández University, Avda. Universidad s/n, Elche 03202, Spain; vgaliano@umh.es; 3 CIBER, Fisiopatología de la Obesidad y la Nutrición, CIBERobn, Instituto de Salud Carlos III., Palma de Mallorca 07122, Spain (CB12/03/30038)

**Keywords:** marine natural product, invertebrate, cancer, virtual screening

## Abstract

Marine secondary metabolites are a promising source of unexploited drugs that have a wide structural diversity and have shown a variety of biological activities. These compounds are produced in response to the harsh and competitive conditions that occur in the marine environment. Invertebrates are considered to be among the groups with the richest biodiversity. To date, a significant number of marine natural products (MNPs) have been established as antineoplastic drugs. This review gives an overview of MNPs, both in research or clinical stages, from diverse organisms that were reported as being active or potentially active in cancer treatment in the past seventeen years (from January 2000 until April 2017) and describes their putative mechanisms of action. The structural diversity of MNPs is also highlighted and compared with the small-molecule anticancer drugs in clinical use. In addition, this review examines the use of virtual screening for MNP-based drug discovery and reveals that classical approaches for the selection of drug candidates based on ADMET (absorption, distribution, metabolism, excretion, and toxicity) filtering may miss potential anticancer lead compounds. Finally, we introduce a novel and publically accessible chemical library of MNPs for virtual screening purposes.

## 1. Introduction

Cancer is defined as “a group of diseases characterized by the uncontrolled growth and spread of abnormal cells” and is one of the deadliest diseases globally. Cancer represents the second most common cause of death in Europe and USA after cardiovascular diseases according to *Cancer Facts and Figures of 2016*, a publication distributed by the American Cancer Society [1], and data extracted in October 2016 from Eurostat-Statistics Explained web. According to the same source, in the EU, the most frequent cancers are colorectal, breast, prostate and lung cancers. The predominant cancer type changes as a function of sex. American Cancer Society estimates that the cancer incidence rate is 20% higher in men than in women. Additionally, while breast cancer is the most common in women, lung cancer is more predominant in men. The second most frequent cancer in both men and women is colorectal cancer. Although significant advances are being made against cancer, this disease remains a key public health concern and a tremendous burden on European and American societies [2]. Every year, the American Cancer Society collects and compiles the most recent surveillance and epidemiology data about cancer (incidence, mortality and survival). In 2017, 1,688,780 new cancer cases will emerge, and 600,920 cancer deaths are projected to occur in the United States [3].

Recently, many advances have been made in the development of surgical procedures, radiotherapy and chemotherapeutic agents [4], including the case of combining chemotherapy and hormone therapy with immunotherapy [5]. One of the main problems that needs to be overcome in cancer treatment is that a tumor should be considered as a heterogeneous multiple cell subpopulation [6]. Another aspect to take into account is the development of resistant phenotypes, which include cytotoxic resistance to anticancer compounds and/or resistance to pro-apoptotic stimuli. All these factors must be considered to develop effective therapies for cancer. Despite all efforts to prevent oncological disorders and to develop new therapies, the cancer rate persists worldwide. Thus, there is an increasing emphasis on strategies to maximize tumor control, prolong survival, minimize chemotherapy side effects and improve quality of life for patients [7]. From a research point of view, this situation also demands the development of new drug discovery strategies and molecules.

Thirty years ago, the main approach of the pharmaceutical industry toward drug discovery was based on combinatorial chemistry (chemical synthetic methods to produce a large number of compounds in a simple process) [8]. By contrast, in the last few years, a new trend on drug discovery has been focused on natural products. Natural products are one of the main sources of compounds for drug discovery and have demonstrated considerable potential in the biomedical field. At least one-third of the current top twenty drugs on the market are derived from a natural source, mainly plants, and approximately 50% of the marketed drugs are classified as naturally derived or designed on the basis of natural compounds [9,10]. In contrast, only one of 5000–10,000 of the new synthetic molecules in development becomes a commercial pharmaceutical drug due to toxicity discovered in the clinical phases. Moreover, meta-analyses have shown that studies sponsored by pharmaceutical companies are several times more likely to report positive results. All these factors are promoting an abundance of scientific studies focused on the putative beneficial effects of natural compounds on human health.

First, it is postulated that natural molecules have been evolutionarily selected to bind to biological macromolecules [11]. Second, these natural molecules, which exhibit unique structures, can serve as lead compounds for the development of new analogues. These factors may be the causes for the high dependence of drug discovery on the continuous supply of novel natural products. Recent advances in synthetic methodology and strategy are overcoming the barriers represented by the structural complexity of many natural products [12]. For example, in the field of antibiotics, resistant strains of pathogenic bacteria are increasing in number, and new compounds to combat these bacterial pathogens are needed. Only a few antibiotics have been developed in the past few decades after generations of synthetic tailoring, but in most cases, they share a common core structure, or scaffold. Multidrug resistance and the latest generation of pathogens suggest the necessity of discovering new chemical structures [11].

The production of metabolites occurs in all organisms as a result of the intrinsic reactions in the body induced by exogenous stimuli or substances [13]. Metabolites can be intermediate or end products of metabolism, and these molecules are transformed into new ones by cascades of enzymatic reactions, which include the processes involved in anabolism, catabolism and excretion of molecules from the body [14]. Because of all these reactions, a set of small molecules, such as metabolic intermediates, hormones and other signaling molecules, are produced, i.e., the metabolome. The general classification of these biomolecules considers two types of metabolites: primary metabolites, which are involved at the cellular level and are essential for life (embryogenesis, cell division, proliferation, differentiation, growth, development, and reproduction) and secondary metabolites, which participate in the homeostasis and natural defense of organisms [15,16].

Natural products, especially secondary metabolites from terrestrial plants and microbes, have been a traditional source of drug molecules for many years (for example, the discoveries of aspirin, morphine and penicillin have led to an obvious “before” and “after” in terms of expectations and quality of life). Even in the area of cancer, products such as adriamycin or paclitaxel (Taxol) are used daily for the treatment of certain tumors [17]. The marine ecosystem offers unlimited sources of new isolable bioactive compounds with diversified chemical structures, which are considered potent sources for drug discovery [18]. Refinements in technologies (like scuba diving or marine prospection), along with the development of new analytical technologies, spectroscopy and high-throughput screening methods, have increased interest in unexplored MNPs.

Marine pharmacology is a new discipline that explores the marine environment searching for potential pharmaceuticals. In the last two decades, a large screening of marine compounds has been conducted, and a wide range of activities, such as antiviral [19] antibacterial [20], antifungal [21], antiparasitic [22], antitumor [23] and anti-inflammatory [24], have been reported. Accordingly, marine compounds are becoming an option to be developed into ingredients for the cosmetic, pharmaceutical and food industries [25]. The marine chemical structures could also become an option for the treatment of drug-resistant infections [26] by offering new model structures. This hypothesis is also transferable to new anticancer drug development. Molecular scaffolds derived from marine organisms exhibit more novelty than terrestrial agents. A comparative analysis reported that 71.02% of molecular scaffolds in the Dictionary of MNPs were used only by marine organisms [27].

The marine environment represents approximately 70% of the earth’s surface, and a huge biodiversity has been found, consisting of up to 36 phyla [28]. There is a hypothesis that high taxonomic diversity is correlated with a wide chemical diversity of natural products [29]. This great diversity of secondary metabolites affords marine organisms with a chance of survival in unfavorable conditions [30,31], and consequently, this diversity offers an abundant source of drugs that could be potential candidates for disease treatments. This is the reason why marine organisms represent a promising source of bioactive molecules [32]. Efforts to exploit this biochemical biodiversity have only just begun, and it is estimated that 18% of MNPs have been discovered so far compared with products of terrestrial origin [33].

**Figure 1:**
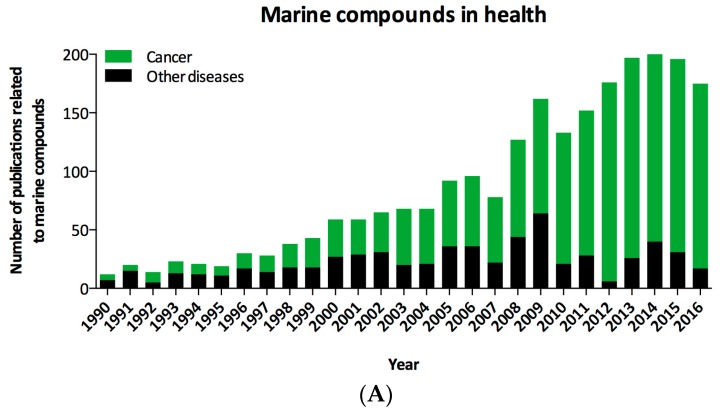

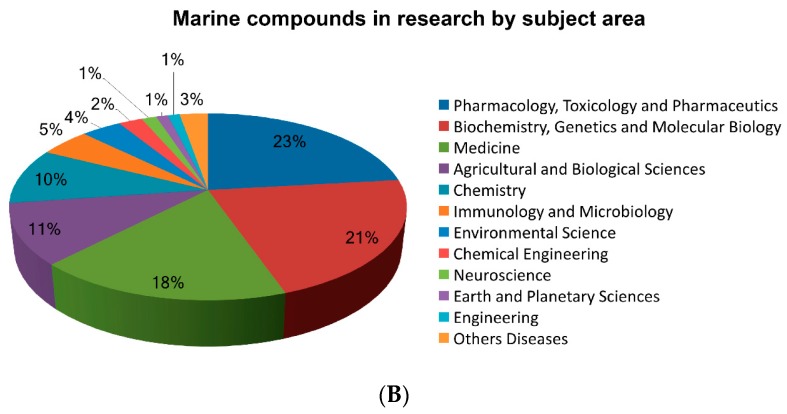
(**A**) The number of scientific publications about marine compounds displays an upward trend in the last twenty years, especially in the field of cancer; (**B**) Relevance of marine compounds by subject area. Data have been obtained from PubMed and Scopus for English language publications published without start date restrictions up to January 2017.

In the history of the use of medicines, references to marine-based drugs are scarce. However, it is known that for thousands of years, ointments, concoctions and cataplasms of algae and marine muds have been used for treating endless diseases, especially in traditional Chinese and Japanese medicines [34]. In 1900, kainic acid, which was obtained from extracts of the seaweed *Digenea simplex*, was the first product of marine origin commercialized and was used as an insecticide and anthelmintic [35]. However, it was not until 1950 that the first drugs from sponges and marine microorganisms were identified. Spongothymidine and spongouridine, the first compounds isolated from the sponge *Cryptotheca crypta*, were obtained by chance [36]. An increasing number of documents about marine compounds with regard to human health have been published in the last twenty years (Figure 1A) in many different areas of knowledge (Figure 1B). To date, eight drugs isolated from marine organisms have been approved for different purposes (cytarabine, vidarabine, ziconotide, omega-3 acid ethyl esters, trabectedin, eribulin mesylate, brentuximab vedotin and iota-carrageenan) [2]. Five of these compounds are obtained from marine invertebrates and have been approved for use as pharmaceutical drugs in cancer treatment. These compounds are summarized in Table 1.

The vast majority of animals living in the ocean fall into the category of invertebrates, and most of the scientific studies to date related to marine drug discovery are focused in these organisms. In fact, marine invertebrates, which lack a backbone (vertebral column), are identified as one of the foremost groups of biological organisms in terms of rich diversity and distribution [42]. Invertebrates are considered a ubiquitous and highly taxonomically diversified group since they can be found from the foreshore to the abyssal zone. Their major phyla include Porifera, Cnidaria, Annelida, Bryozoa, Mollusca, Arthropoda and Echinodermata [43]. Some invertebrate phyla have only one species, while others include more than 85% of all described animal species and consist of over a million species [43]. The most common marine invertebrates are from the Porifera, Cnidaria, Mollusca, Arthropoda and Echinodermata phyla and have provided a significant number of natural products with pharmacological properties, some of which are already in clinical trials [44]. In response to exposure to extreme and changing habitats, these organisms produce a wide variety of secondary metabolites that cannot be produced in other organisms. In addition to the number of unique natural products generated by invertebrates, the diversity of the group makes them a singular source of bioactive compounds [33]. At present, the various bioactive compounds from different invertebrates (cone snails, soft corals, sponges, sea squirts, marine worms, bryozoans, sea slugs and other marine organisms) have been recognized as an important source of antitumor compounds [45]. A study of 2011 by Hu et al. showed that approximately 75% of the 20,000 MNPs described to date are derived from marine invertebrates [46]. Therefore, it is expected that in the future, bioprospecting efforts will continue to target marine invertebrates. In 2012, Leal et al. [47] compiled scientific studies about existing MNPs and classified them according to phyla of their invertebrate source. They found that approximately 80% were into the phyla Porifera (47.1%) and Cnidaria (33.5%). The rest of them were in the phyla Echinodermata (7.4%), Chordata (6.0%), and Mollusca (5.0%). They also compared the MNPs by chemical group and demonstrated that in the Porifera or Cnidaria phyla, a particular chemical group was not always prevalent through the same phylum [47].

Typical compounds with health benefits obtained from invertebrates include polyunsaturated fatty acids, peptides, proteins, polysaccharides, polyphenols, saponins, sterols, minerals, and other bioactive compounds such as pigments (e.g., carotenoids) [48]. To date, more than 20,000 novel chemicals have been found from marine sources, and that number is rising every year [33]. In recent years, these marine secondary metabolites have yielded a considerable number of drug candidates, most of which are still in preclinical or early clinical development, with only a limited number already on the market [49]. Table 2 contains the most relevant examples of MNPs in clinical and preclinical phases.

The purpose of this article is to review the research literature published since 2000 in the field of marine antitumor pharmacology and propose new *in silico* strategies to accelerate drug discovery. In this sense, novel antitumor marine compounds from invertebrates grouped by their chemical structures, their putative mechanisms of action and their use in preclinical or clinical cancer studies are discussed. Moreover, we have analyzed the discrepancy in the results from a comparison between the properties of drug candidates filtered *in silico* from a particular library that obeys ADMET rules and the properties of the 168 most promising anticancer compounds from the “Genomics of Drug Sensitivity in Cancer (GDSC) database”. A new library of MNPs for *in silico* and analytical purposes is also presented (http://docking.umh.es/chemlib/mnplib).

## 2. Chemical Classification of Marine Bioactive Compounds

Although several classifications have been made [46,50], taking into account their chemical structures, the most common chemical classes of MNPs are alkaloids, polyketides, terpenes, peptides, and carbohydrates. This section gives a brief description of the principal characteristics of the different classes of marine-sourced compounds.

### 2.1. Alkaloids

Alkaloids are a highly diverse group of widely distributed compounds. Pelletier et al. [48] defined alkaloids as “cyclic organic compounds containing nitrogen in a negative oxidation state which is of limited distribution among living organisms”. There are various classifications of alkaloids in terms of their chemical structure, biological activity, biosynthetic pathway, and composition as heterocyclic or nonheterocyclic compounds [51]. Kumar et al. [52] classified alkaloids into seven subclasses: pyridoacrine alkaloids, indole alkaloid, pyrrole alkaloids, isoquinoline alkaloids, guadinine alkaloids, aminoimidazole alkaloids, and sterol alkaloids [53]. Alkaloids have been isolated from marine organisms such as sponges, tunicates, anemones, and mollusks, all of which are characterized bright colors and patterns, which are often related to alkaloids [54]. Alkaloids are attributed a wide range of biological activities, including antifouling [55], cytotoxic [56], antileukemic [57], antimalarial [58] and antimicrobial [59].

### 2.2. Polyketides

Polyketides are natural metabolites that comprise a highly diverse class of chemical structures. Compounds in this class include macrolides, polyethers, polyols and aromatic compounds. This class is often highly oxygenated [53] and contains multiple β-hydroxyketone or β-hydroxyaldehyde functional groups. Polyketides are complex organic compounds similar to fatty acids: first, because both are synthesized by the decarboxylative condensation of malonyl-CoA and other acyl-CoAs; however, in polyketides, more than one monomer type may be used to construct different sized aromatic groups or reduced chains. Second, polyketides and fatty acids are associated with a wide variety of essential cellular functions; however, polyketides are more complex in their biosynthetic routes [60]. These metabolites are isolated from sponges, ascidians, soft corals and bryozoans [33] and can be produced by commensal or symbiotic bacteria [61]. Polyketides possess wide-ranging biological activities, including antibiotic, anticancer, antifungal, antiparasitic and neurotoxic effects [53].

### 2.3. Terpenes

Terpenes are the final products from biosyntheses involving a five-carbon isoprene structure. Depending on the number of units, they can be classified as monoterpenes, sesquiterpenes, diterpenes, sesterterpenes, triterpenes (steroids), and tetraterpenes (carotenoids) [53,62]. Several groups of marine organisms produce terpenes, which exhibit biological activities such as cytotoxic, antiproliferative, antifouling, antifungal, and antimicrobial activities [63].

### 2.4. Peptides

Peptides are sources of nitrogen and amino acids ranging in size from 2 to 20 amino acids residues and are related to numerous potential physiological functions. Bioactive peptides can be protein fragments that acquire functionality when liberated from the parent protein [64]. The first activity assigned to a peptide was neurotoxicity; however, at present, they are associated with other functions, such as cardiotonic, antiviral and antitumor, cardiotoxic and antimicrobial activity [65]. These functions, in addition to their excellent binding properties, low off-target toxicity, and high stability, make peptides promising molecules for the development of new therapeutics [66]. Approximately 60% of described natural products belong to peptide family [67]. Peptides are present in many marine species, and the extensive research that has been conducted on them has shown that they most often found in sponges [65], as occurred with polyketides, peptides can be also produced by commensal or symbiotic bacteria or fungi.

### 2.5. Carbohydrates, Glycosides and Others

The major class of carbohydrates that can be isolated from marine organisms are polysaccharides [53], but it is also possible to obtain low molecular weight glycosylated oligosaccharides, usually from sponges and tunicates [68]. Polysaccharides are a diverse class of macromolecules comprising polymers of monosaccharides bonded with glycosidic residues and exhibiting a wide structural diversity [69]. These versatile compounds are used extensively in pharmaceuticals, including in gel production, drug delivery systems, wound healing, tissue engineering, and blood dialysis membranes. In addition, they have displayed functions such as antimutagenic, antitumorigenic, hypocholesterolemic, and anticoagulant activities [70]. Marine oligosaccharides are generated by the hydrolysis of marine polysaccharides. Normally, oligosaccharides contain between 10 to 12 monosaccharide units, but certain oligosaccharides have 30 or more units, such as some rhamnogalacturonans [71]. Oligosaccharides have complex and heterogeneous chemical structures, and this is correlated with the diverse biological actions in which they are involved [72]. Despite their biological relevance, there are no studies about the anticancer activity of carbohydrates of marine origin.

Glycosides are considered a class of carbohydrates. These molecules have a sugar bound to another functional group through a glycosidic bond. They have two parts, the sugar and the aglycone chemical group (a terpene, flavonoid, coumarine or other natural molecule) [73].

Glycosaminoglycans are molecules with a linear and complex carbohydrate structure [74]. These molecules are present on all animal cell surfaces and display considerable sequence heterogeneity, which is responsible for their highly specific interactions with other macromolecules [75].

Nucleoside analogues such as cytarabine and gemcitabine are compounds that have similar structures to cytosine and that inhibit DNA synthesis by being incorporated into nascent DNA chains, interfering with chain elongation and promoting abnormal fragment ligation.

## 3. MNP-Based Drugs that Are Approved or in Ongoing Clinical Trials

Some MNPs have already provided promising results in their preclinical phases and have being promoted to clinical trials or even approved by regulatory agencies. This section provides updated information on the most significant examples of currently approved drugs (summarized in Table 1) and those that are still in clinical trials (summarized in Table 2), grouping these compounds according to their chemical structure and giving details about their origins and their potential mechanisms of action.

**Figure 2:**
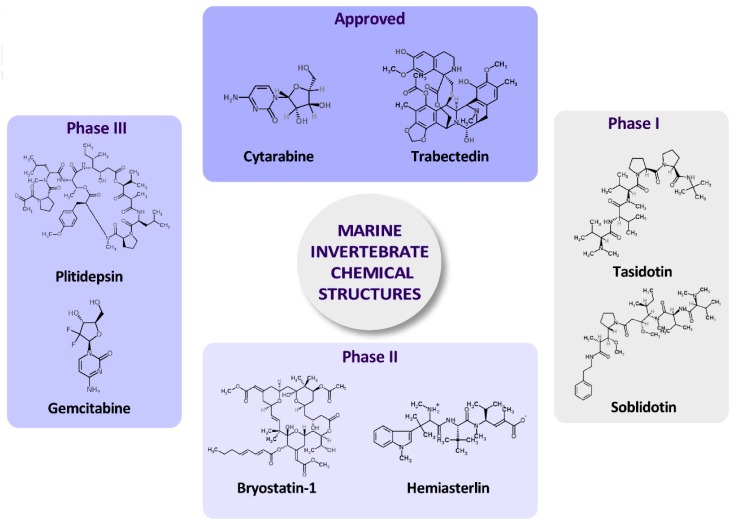
Chemical structures of selected marine invertebrate compounds that are either approved or in clinical trials.

Trabectedin (Yondelis^®^, ET-743) (Figure 2), a semisynthetic tetrahydroisoquinoline alkaloid that was originally derived from the marine tunicate *Ecteinascidia turbinata,* was the first marine anticancer agent approved in the European Union for patients with soft tissue sarcoma (Table 1) [39,90]. PM1004, or Zalypsis^®^, is related to jorumycin, a natural alkaloid isolated from the skin and mucus of the Pacific nudibranch *Jorunna funebris* [91,92] and also found in sponges and tunicates [92]. This compound is in phase II clinical studies (Table 2) because of its putative chemotherapeutic activity against solid human tumors and hematological malignancies (Ewing sarcoma, urothelial carcinoma, endometrial and cervical cancer and multiple myeloma) [83,93].

Among clinically approved polyketides, eribulin mesylate (E7389) (Table 1), which was originally extracted from the marine sponge *Halichondria okadai,* is an analogue of halichondrin B that acts as a non-taxane microtubule dynamics inhibitor. The FDA approved E7389 for the treatment of liposarcoma and breast cancer in 2010 [94]. Discodermolide (Table 2) is a polyketide in phase I/II trials isolated from the marine sponge *Discodermia dissoluta* that acts as a microtubule interfering agent, binding microtubule bundles, disrupting mitotic spindles and inducing cell cycle arrest at the G2/M phase [95]. Bryostatin 1 is a polyketide (Table 2 and Figure 2) isolated from the bryozoan *Bugula neritina* and derived from sponges and tunicates. This compound has been proven to be active against multiple carcinomas [96] and is currently undergoing two phase I trials for assessment as a treatment for Alzheimer’s disease and cancer [37] (Table 2).

Although there are no compounds from the terpene family in clinical trials related to their application in cancer treatment, there are data on the use of these compounds in other diseases. For example, the pseudopterosins (Table 2), which are diterpene glycosides from the soft coral *Pseudopterogorgia elisabethae*, showed potent activity as anti-inflammatory and wound-healing agent in a phase II clinical trial [97]. Moreover, Mayer et al. found that both pseudopterosin E and pseudopterosin A were effective at reducing edema when administered topically [98].

The most representative pharmaceutical products derived from marine peptides are ziconotide (Table 1) and brentuximab vedotin (SGN-35), which are a natural marine peptide and a peptide derivative, respectively, that have reached the market [99]. Brentuximab vedotin (Table 1) is an approved peptide derivative manufactured by chemical synthesis. This compound is an antibody-drug conjugate with a strong antimitotic capacity that targets the cell membrane protein CD30. It was approved by the FDA in 2011 for the treatment of Hodgkin and systemic anaplastic large-cell lymphoma [100].

Plitidepsin is a compound that features a peptide chemical structure (Table 2) and is currently in clinical phase III trials [101]. This marine cyclic depsipeptide (a polymeric compound with peptide and ester linkages), also known as Aplidin^®^, (Figure 2) is isolated from the Mediterranean tunicate *Aplidium albicans* [102] and is now accessible via chemical synthesis. Plitidepsin is in phase III clinical development for several neoplasias, including breast, melanoma and non-small-cell lung cancers [103]. Plitidepsin induces dose-dependent cell cycle arrest in cultured cells from solid tumors and apoptotic process through the activation of c-Jun N-terminal kinase (JNK) [103]. In hematological cancer cells, plitidepsin activates the intrinsic and extrinsic apoptotic pathways [104,105] at nanomolar concentrations.

Soblidotin (TZT-1027) (Figure 2), which is in phase II trials, is an microtubule active drug derived from the antimitotic dolastatin-10, which was isolated from the sea hare *Dolabella auricularia*, that specifically damages tumor vasculatures by exerting a considerable antivascular effect along with an excellent cytotoxic effect [106]. Tasidotin (Table 2 and Figure 2), or synthadotin (ILX-651), is a water-soluble dolastatin pentapeptide derivate [107] with microtubule targeting activity in advanced solid tumors [107] and is also in phase II trials. Elisidepsin (PM02734, Irvalec^®^) and glembatumumab vedotin (Table 2) are also in phase II clinical trials. Elisidepsin, a synthetic cyclic depsipeptide belonging to the Kahalalide family of compounds [108], is derived from a sacoglossan sea slug species, *Elysia rufescens.* Glembatumumab vedotin is a peptide in phase II (Table 2) that was isolated from mollusks and cyanobacteria and is currently synthesized to treat breast cancer and melanoma [109]. Pinatuzumab vedotin and Tisotumab vedotin, both of which were isolated from mollusks and cyanobacteria, are also in phase I trials. Pinatuzumab vedotin has activity against non-Hodgkin lymphoma and chronic lymphocytic leukemia [110]. Tisotumab vedotin (HuMax^®^-TF-ADC) is an antibody-drug conjugate that has inhibited tumor growth in preclinical experiments [111]. Hemiasterlin (E7974), which is also in phase I trials (Table 2 and Figure 2), is a cytotoxic tripeptide from the marine sponges *Hemiasterella minor*, *Cymbastela* sp. and *Auletta* sp. [112] consisting of three sterically congested amino acids. The compound showed a potent antimitotic effect related to the inhibition of tubulin polymerization by binding to the Vinca alkaloid binding site [113]. Marchetti et al. [114] have developed a hybrid drug by conjugating two tubulin inhibitors, one of which was hemiasterlin. This derivative possessed stronger and more potent anti-tubulin activity against human Caucasian ovary adenocarcinoma SKOV3 cells [114]. Taltobulin (HTI286), another hemiasterlin derivative, is also in phase I clinical trials [84].

Cytarabine (Ara-C) (Table 1, Figure 2) is a synthetic compound derived from spongothymidine, which was isolated from the marine sponge *Cryptotheca crypta*. This compound is a nucleoside that has already been approved and commercialized and that has mainly been used in leukemia [115]. Gemcitabine (Figure 2) is a nucleoside analogue (specifically, a fluorinated derivative of cytarabine) in phase II and III clinical development [77,116] and is used in various carcinomas, such as non-small cell lung cancer, pancreatic cancer, bladder cancer and breast cancer [117].

Finally, dacinostat (LAQ824) (Table 2 and Figure 2) is a synthetic hydroxamic acid derivative in phase I trials that inhibits histone deacetylases (which are validated targets for anticancer therapy) at nanomolar concentrations [118].

## 4. MNPs under Research or in Preclinical Stages Classified by Cancer Molecular Targets

**Figure 3:**
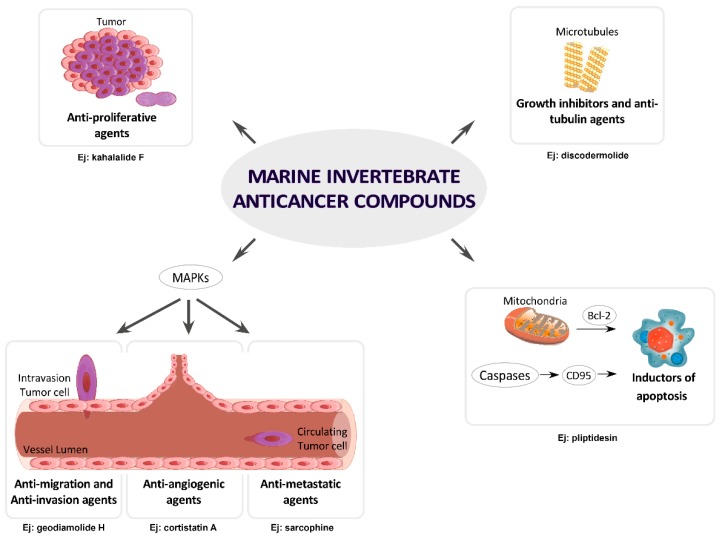
Major molecular targets of marine compounds known to modulate different hallmarks of cancer.

In this part of the review, compounds under active research or in preclinical phases are summarized according to the ability of these natural products to modulate one or more hallmarks of cancer and become promising anticancer drug candidates. This section presents an overview of the most relevant compounds from 2010 to 2017. Before starting, it must be considered that normal cells become cancer cells through a wide range of genetic changes, and this process generates many different types of cancer [119]. However, most cancer types share similar characteristics, and these must be studied to progress anticancer drug discovery and cancer treatment. Hanahan and Weinberg identified six major targets in human tumors: self-sufficiency in growth signals, insensitivity to growth-inhibitory (antigrowth) signals, evasion of programmed cell death (apoptosis), limitless replicative potential, sustained angiogenesis, and tissue invasion and metastasis [120]. This part of the review focuses on marine compounds that play a role in some of the hallmarks described by Hanahan and Weinberg. Then, we have classified MNPs as growth inhibitors and anti-tubulin agents, inductors of apoptosis and autophagy, and anti-angiogenic, anti-migration, anti-invasion and anti-metastatic agents. In addition, due to their relevance in signal transduction pathways, a supplementary family that includes inhibitors of proliferation and of mitogen-activated protein kinases (MAPKs) are also included. All these categories are shown in Figure 3.

### 4.1. Growth Inhibitors and Anti-Tubulin Agents

Microtubules are cytoskeletal fibers composed of tubulin subunits. These structures are the key components of the cytoskeleton and are essential in all eukaryotic cells because of their functions, namely, the maintenance of cell shape, the transport of vesicles and cell signaling molecules, mitochondria homeostasis, motility and organelle distribution [121]. In addition, the microtubule system plays an important role in mitosis and cell division, making it an important target for anticancer drugs [122]. Microtubules are composed of heteropolymers of α-, y- and β-tubulin in a head-to-tail arrangement that form dimers. These dimers polarize into 13 parallel protofilaments, which form a rigid hollow structure. Because both α- and β-tubulin dimers bind to GTP before assembling into microtubules, the equilibrium between the dimeric state and the polymeric state is regulated by dynamics that are dependent on the hydrolysis of the GTP nucleotide. GTP-bound heterodimers are more likely to polymerize, whereas those bound to GDP shift the equilibrium toward the free species. GTP is essential for the stability of the microtubules and the polymerization-depolymerization equilibrium. Microtubules and free tubulin dimers exist in a highly dynamic equilibrium, which is very sensitive to external factors [123]. Antitumor drugs can disrupt the microtubule equilibria, inhibiting the mitotic spindle and acting as antimitotic agents by causing mitotic arrest [124]. Microtubule-targeted antimitotic drugs can be divided into microtubule-destabilizing agents and microtubule-stabilizing agents (Figure 3). The Vinca alkaloids (vinblastine, vincristine, vinorelbine, vindesine and vinflunine), cryptophycins, halichondrins, estramustine, colchicine and combretastatins are microtubule-destabilizing agents because they inhibit microtubule polymerization at high concentrations. The microtubule-stabilizing agents stimulate microtubule polymerization and include paclitaxel, docetaxel, the epothilones and discodermolide [125,126]. Microtubule-targeting agents are important anticancer drugs used in the treatment of breast, ovarian, and lung cancer [127]. The main MNPs associated with this category are listed below.

Dolastatin-10 was isolated from the marine mollusk *Dolabella auricularia* and the marine cyanobacterium *Symploca* sp. [128]. This compound is a linear pentapeptide containing several unique amino acid subunits and is one of the most potent members of a large class of peptides [129]. Dolastatin-10 was compared to other antimitotic drugs (vinblastine, maytansine, rhizoxin, and phomopsin A) in terms of their ability to interfere with the binding of Vinca alkaloids to tubulin. The polymerization inhibition effect of dolastatin 10 was found to be even higher than that of vinblastine [129]. As mentioned above, there are two drugs based on dolastatin-10 in clinical trials: soblidotin and tasidotin (Table 2).

Vitilevuamide, a bicyclic 13 amino acid peptide isolated from two marine ascidians, *Didemnum cuculiferum* and *Polysyncranton lithostrotum* [130], had a cytotoxic effect at nanomolar concentrations on several human tumor cell lines. This compound exhibited potent inhibition of tubulin polymerization, displaying activity in vivo against P388 lymphocytic leukemia and showing an interaction with the GTP binding site, which was also weakly affected by the presence of vitilevuamide.

Some peptides from marine sources, such as diazonamide A [131] (isolated from the marine ascidian *Diazona angulata*) [132] and scleritodermin A [133] (isolated from the sponge *Scleritoderma nodosum*), have shown potent disruption of tubulin polymerization in different cancer cells and have a unique binding site on tubulin from the *Vinca* alkaloids [134].

Spongistatins, macrocyclic lactones containing six pyran rings that were isolated from sponges in the genus *Hytrios*, has exhibited a potent cytotoxic effect on solid tumors and thus are in phase I clinical trials. These compounds are also noncompetitive inhibitors of vinblastine and dolastatin binding [135]. At nanomolar concentrations, spongistatins exhibit potent activity by inhibiting microtubule organization and preventing mitotic spindle formation in human breast cancer cells [135].

Taltobulin (HTI-286) (Table 2) is a synthetic analogue of the naturally occurring tripeptide hemiasterlin (isolated from the sponge *Hemiasterella minor*) with potential antimitotic and antineoplastic activities. This compound has advanced to clinical trials due to its ability to inhibit tubulin polymerization in a similar manner to colchicine and cause cell cycle arrest at the G2/M phase, blocking cell division and apoptosis [136].

Peloruside A, a macrolide (polyketide) isolated from the marine sponge *Mycale hentscheli*, binds to a distinct, non-taxoid binding site on tubulin. This compound was tested in a human breast adenocarcinoma cell line (MCF7) stably expressing GFP-α-tubulin, and peloruside A at nanomolar concentrations was found to be able to inhibit the growth rate and change the length of microtubules [137] in a concentration-dependent manner. Zampanolide is another marine natural macrolide that belongs to the family of microtubule-stabilizing cytotoxic agents and was originally isolated in 1996 from the marine sponge *Fasciospongia rimosa* [138]. This compound has a highly unsaturated, 20-membered macrolide ring and an N-acyl hemiaminal side chain. Zampanolide was characterized as a cytotoxic agent that promoted tubulin assembly and blocked cells in G2/M phase of the cell cycle [139]. In recent studies, it has been confirmed as a microtubule-stabilizing agent that covalently reacts with the taxane luminal site in both tubulin α,β-heterodimers and microtubules [140].

Dictyostatin, an antimitotic macrolide (polyketide) isolated from the sponge *Corallistidae* sp., demonstrated cytotoxic properties in human cancer cells at nanomolar concentrations with a Taxol-like mechanism of action, binding to tubulin and promoting microtubule assembly [141,142]. As mentioned above, Discodermolide (Table 2) is a polyketide already in clinical trials. In addition to its activity under study, this compound exerted antiproliferative action against paclitaxel-resistant human tumor cells that presented β-tubulin mutations [143]. Several hybrid compounds of discodermolide and dictyostatin have been developed and found to maintain their antiproliferative activity against several taxane-resistant cell lines [144,145]. Laulimalide is a macrolide isolated from the marine sponge *Cacospongia mycofijiensis* and has the ability to inhibit cell proliferation with anti-tubulin activity by promoting the assembly of the microtubules and stabilizing them in a Taxol-like way. However, this compound has a different binding site, which is placed on two adjacent β-tubulin units between the tubulin protofilaments of a microtubule [146].

### 4.2. Inductors of Apoptosis and Autophagy

In an organism, programmed cell death is a terminal point for cells and is involved in various process, such as morphogenesis, maintenance of tissue homeostasis, and elimination of damaged cells. Dysfunction of programmed cell death and accumulation of errors can allow malignant cells to survive and disseminate. Many studies have categorized programmed cell death into three types: apoptosis, autophagy and a variant form of apoptosis called necroptosis [147,148]. This section of the review is focused on marine compounds that lead the apoptosis and autophagy of cancer cells.

Apoptosis involves the genetically programmed elimination of cells [149] and is a fundamental process associated with development, physiology and homeostasis. Any alteration in the balance of pro- and anti-apoptotic signals can lead to a variety of pathological conditions, including cancer [150].

In mammals, the most recent research indicates the existence of two major signaling systems that result in caspase activation and produce apoptosis (Figure 3). These pathways are the intrinsic or mitochondrial death [151] and the extrinsic or death receptor [152] pathways. Both are related to the cleavage of caspase-3 and produce fragmentation of DNA, degradation of cytoskeletal and nuclear proteins, cross-linking of proteins, formation of apoptotic bodies, expression of ligands for phagocytic cell receptors and finally uptake by phagocytic cells [153]. The intrinsic pathway is stimulated by intracellular stress such as radiation, growth factor reduction, decreased cytokines and cytotoxic drugs. This pathway is controlled by the Bcl-2 family of proteins [154], which release cytochrome c (Cyt c) from the mitochondria to the cytosol and interact with APAF-1 to generate the apoptosome, a molecular platform for caspase 9 activation, which consequently activates caspases 3 and 7. The extrinsic pathway does not involve the mitochondria and is stimulated by cell-surface death receptors such as CD95 (Apo-1 or Fas), TRAIL, and tumor necrosis factor receptor 1.

Autophagy, an evolutionarily conserved catabolic process, is a cellular degradation pathway for the clearance of damaged or superfluous proteins and organelles [155]. Autophagy is often activated by reactive oxygen species (ROS) accumulation, hypoxia, nutrient deprivation, drug stimuli or endoplasmic reticulum stress (ERS). The main proteins involved in autophagy are the mammalian target of rapamycin complex 1 (mTORC1), phosphatidylinositol 3 kinase (PI3K), kinase AKT (AKT), Beclin-1 and p53 [156]. The PI3K/AKT pathway is the upstream target of the kinase mTOR. When PI3K and AKT are activated, autophagy is inhibited by the activation of mTOR. By contrast, inhibition of mTOR would activate autophagy [157].

Jasplakinolide (jaspamide), a cyclic depsipeptide isolated from the marine sponges *Jaspis johnstoni* and *J. splendens* [158,159], induces apoptosis in Jurkat T cells [33]. Jaspamide was found to induce apoptosis not only by activating caspase 3 and decreasing Bcl-2 protein expression but also by increasing Bax expression levels. Several new analogues have been isolated (B, C, V, etc.) from jasplakinolide, and all of them possess anticancer activity, as determined using NCI 60 cells [160]. Dolastatins 10 and 15 are potent linear pentapeptides isolated from the marine sea hare *Dolabella auricularia* [128,161]. Dolastatin 10 and bryostatin 1 (a polyketide isolated from the bryozoan *Bugula neritina*) can modulate the Bcl-2 and p53 oncoproteins in human diffuse large-cell lymphoma. In one study, the expression of Bcl-2 by human diffuse large cell lymphoma WSU-DLCL2 cells decreased when the cells were treated with bryostatin 1 and dolastatins 10 and 15 [162].

CS5931, a polypeptide isolated and purified from the sea squirt *Ciona savignyi*, has shown potent anticancer activity and induced apoptotic cell death in ileocecal colorectal adenocarcinoma (HCT-8) cells in a dose-dependent manner [163]. One study showed that in human carcinoma colorectal cells exposed to this compound, CS5931 exerted strong cell cycle arrest at the G2/M phase and a sub-G1 peak phase and induced release of Cyt C and activation of caspase 9 and 3. Mere 15 is a linear polypeptide isolated from the bivalve *Meretrix meretrix*. This compound exhibited in vivo activity against the growth of a human lung adenocarcinoma A549 xenograft in nude mice in a dose-dependent manner. This compound was also able to induce cell cycle arrest in G2/M phase followed by apoptosis. This apoptosis was characterized by the cleavage of procaspases 9 and 3, membrane blebbing, loss of mitochondrial membrane potential with subsequent Cyt C release, externalization of phosphatidylserine, condensation of chromosomes and fragmentation of DNA [164]. The didemnins are a family of depsipeptides isolated from the tunicate *Trididemnum solidum* with antitumor, antiviral and immunosuppressive activities. The most characterized compound of this family is didemnin B, a branched N-methylated cyclic peptide, which selectively induces rapid and extensive apoptosis in a panel of breast cancer and colon cancer cell lines [165].

Monanchocidin A, a novel guanidine alkaloid isolated from the marine sponge *Monanchora pulchra* [166], features an unprecedented skeleton system derived from a polyketide precursor, (ω-3)-hydroxy fatty acid. This compound shows cytotoxicity against some cancer cell lines, such as human leukemia (THP-1), mouse epidermal (JB6 Cl41), and human cervix epithelioid carcinoma (HeLa) at micromolar levels. However, its mechanism of action has not been elucidated to date [166]. In a recent study, monochocidin A was tested in genitourinary cancer cell lines and showed the capability to affect malignant cell lines more intensively than non-malignant cell lines in a different way from classical apoptosis [167].

Tyrindoleninone, tyrindolinone, 6-bromoisatin and 6,6′-dibromoindirubin are indole derivatives from the Muricidae family of marine gastropods with anticancer properties [168,169,170]. An in vivo study on a rodent model of colon cancer demonstrated the pro-apoptotic properties of crude extracts from the muricid gastropod *Dicathais orbita* [170]. Tyrindoleninone and 6-bromoisatin showed strong cytotoxicity against several colon cancer cells (HT29 and Caco-2) and were able to increase caspase 3/7 expression in both cell lines, inducing apoptosis [171].

The makaluvamines, a class of marine pyrroloiminoquinone alkaloids isolated from sponges in the genus *Zyzzya*, have been reported to possess potent in vitro and in vivo cytotoxicity against several human cancer cell lines. Their activity is related to topoisomerase II inhibition [172,173]. Chen et al. showed that a synthetic makaluvamine analogue (FBA-TPQ) had potent cytotoxic activity against human ovarian cancer A2780 and OVCAR-3 cells and induced apoptosis, G2/M cell cycle arrest and dose-dependent inhibition of OVCAR-3 xenograft tumor growth in female athymic nude mice (BALB/c, nu/nu) [174]. Analogues of the marine pyrrole-2-aminoimidazole alkaloids, i.e., oroidin, clathrodin, and hymenidin, isolated from Agelas sponges exhibited apoptotic-inducing activity in the HepG2 cell line with 25–38% apoptotic cells at 50 µM [175].

Echinoside A and ds-echinoside A, which are glycosylated triterpenes isolated from the sea cucumber *Pearsonothuria graeffei*, showed cytotoxic activity against the P388, A549, MKN-28, HCT-116 and MCF-7 cancer cell lines. Both compounds displayed potent activity in blocking cell cycle progression and induced apoptosis through the intrinsic mitochondrial pathway, with ds-echinoside being the most potent one [176]. Stelletin A is a triterpenoid obtained from the marine sponge *Geodia japonica* that has a cytotoxic effect on murine melanoma B16F10 cells. Its effect is associated with inducing ERS and upregulating unfolded protein chaperone and glucose-regulated protein 78 in a dose-dependent manner. It was also shown to induce autophagy by increasing the levels of the membrane-bound form (LC3II) of autophagosome-associated protein light chain 3 (LC3). Wang et al. isolated the triterpene stellettin B from the marine sponge *Jaspis stellifera*, and this compound caused G1 phase cycle arrest, apoptosis and autophagy in adenocarcinomic human lung adenocarcinoma cells (A549 cells), activity that was associated with a reduction in PI3K-p110 expression and the inhibition of the PI3K/Akt/mTOR pathway [177]. Frondoside A, a glycosylated triterpene isolated from the marine cucumber *Cucumaria frondosa*, has shown anti-migration and invasion activity [178]. In a recent study [179], frondoside A was able to induce cell cycle arrest in metastatic castration-resistant prostate cancer cells through caspase-dependent and caspase-independent apoptosis and to inhibit pro-survival autophagy. In animal trials, this compound inhibited the tumor growth of PC-3 and DU145 cells with a notable reduction in lung metastasis and in circulating tumor cells in the peripheral blood.

Ilimaquinone is a sesquiterpene quinone isolated from the Hawaiian sponge *Hippospongia metachromia* [180]. It is known to induce Golgi membrane fragmentation and is widely used to study the mechanism of vesicular trafficking [181]. This compound was able to promote hypoxia-inducible factor-1 (HIF-1) [182] and to induce G1 phase cycle arrest through upregulation of DNA damage-inducible gene 153 (CHOP/GADD153) in prostate cancer cells [183]. Another study on ilimaquinone and an ilimaquinone analogue (ethylsmenoquinone) showed that these compounds were able to induce G2/M cell cycle arrest and increase apoptosis by caspase-3 cleavage and autophagy; this mechanism was stimulated by both compounds through microtubule-associated protein 1 light chain 3 (LC3) in HCT116 colon cancer cells [184].

Nortopsentins A-C, which are indole imidazole alkaloids isolated from the marine sponge *Spongosorites ruetzleri*, exhibited in vitro cytotoxicity against leukemia P388 cells [185]. Two analogues of nortopsentin, 5-bromo-1-methyl-3-[2-(1*H*-pyrrolo[2,3-*b*]pyridin-3-yl)-1,3-thiazol-4-yl]-1*H*-pyrrolo[2,3-*b*]pyridine (T1) and 3-[2-(5-Bromo-1*H*-indol-3-yl)-1,3-thiazol-4-yl]-7-chloro-1*H*-pyrrolo[2,3-c]pyridine (T2) have shown selective cytotoxic effects on a broad spectrum of human cancer cell lines included in the NCI panel, whereas they did not affect normal intestinal cells. T1 was able to affect the apoptotic cell population by activating the mitochondria-mediated pathway in a dose-dependent manner, inducing cell cycle arrest at the G2/M phase, and inhibiting CDK-1 activity in HCT 116 colon cancer cells [186,187,188]. On the other hand, in the same cell model, T2 seemed to induce antiproliferative effects through apoptosis at high concentrations but effected a massive accumulation of autophagic vacuoles without apparent signs of apoptosis at low concentrations [189].

### 4.3. Inhibitors of Angiogenesis, Migration, Invasion or Metastasis

Angiogenesis is a normal physiological process that not only encompasses the proliferation, migration and morphogenesis of endothelial cells during the development of new blood vessels but also is responsible for providing oxygen and nutrients [190]. This process occurs during organogenesis or in a damaged organ and is involved in wound-healing activity, menstruation and placenta formation during pregnancy. In cancer, angiogenesis provides an opportunity for tumor growth and circulates tumor cells via the blood stream to other organs, inducing metastases [191]. Angiogenesis is controlled by a finely tuned balance of angiogenic inhibitors and stimulators. When angiogenesis is activated, first the established vessel is destabilized, followed by endothelial cell proliferation and migration and new tube formation [192].

The main actors of the angiogenic process are vascular endothelial growth factor (VEGF) and its receptor, VEGFR-2 (Flk-1/KDR). Angiogenesis is also associated with extracellular factors, including interleukin-8 (IL-8), tumor necrosis factor (TNF), fibroblast growth factor-2 (FGF-2), platelet-derived growth factor (PDGF), platelet-derived endothelial cell growth factor (PD-ECGF), angiopoietin-1, transforming growth factor beta-1 (TGF-β1), transforming growth factor alpha (TGF-α) and epidermal growth factor (EGF) [193,194]. Blocking the VEGF-VEGFR-2 pathway and its downstream signaling is another strategy that leads to tumor growth inhibition. VEGF is overexpressed in some cancer cells and is a key factor in initiating the processes of angiogenesis, proliferation, migration, invasion and tube formation in endothelial cells [195].

Metastasis is probably the most serious feature of cancer, and it determinates clinical stages and prognosis [196]. Changes that cause tumor cells to become metastatic are complex and numerous, but some of the molecular targets are well identified. Matrix metalloproteinases (MMPs) are a family of zinc-dependent endopeptidases that play important roles in the degradation of the extracellular matrix and in the invasion and metastasis of tumors. MMPs, integrins, ICAM-1 and the extracellular matrix (ECM) are major factors involved in metastasis. MMP2 and MMP9, which are ECM degradation proteins, are the first actors on metastasis by locally degrading the ECM [197]. The next steps in the journey of a metastatic cancer cell are infiltration of the stroma and vascular tissue and extravasation and invasion of new tissue [198].

Research on the angiogenic and metastatic processes in cancer is mainly focused on compounds that block proteases, endothelial cell migration and proliferation, angiogenic growth factors or matrix proteins on the endothelial cell surface, such as integrins [199]. Compounds that can disrupt or normalize the processes of angiogenesis and/or metastasis are potential drugs for cancer treatment [200], and the main MNPs with these characteristics are listed below.

Geodiamolide H is a cyclodepsipeptide isolated from the Brazilian marine sponge *Geodia corticostylifera* [200]. This compound inhibited the invasion and migration activity of the human mammary Hs578T cell line in a dose-dependent manner (at nanomolar concentrations) in three types of assays: by time-lapse microscopy, in a Transwell^®^ invasion assay and in a scratch-wound closure assay. This effect was attributed to its modification of the actin cytoskeleton of tumor cells; by contrast, normal cells were not affected. Stylissamide X is a proline-rich cyclic octapeptide isolated from an Indonesian marine sponge, *Stylissa* sp. [201]. Although this compound did not substantially modify cell viability at low micromolar concentrations (0.1 to 10 μM), it was able to inhibit the migration of HeLa adenocarcinoma cells in both a wound-healing assay and a chemotaxicell chamber assay. A marine oligopeptide isolated and purified from the gastropod abalone (*Haliotis discus hannai*) had a specific inhibitory effect on MMP-2/-9 and reduced the expression of proteins p50 and p65 in human fibrosarcoma (HT1080) cells in a dose-dependent manner [202].

Suberreamoline A, a bromotyrosine alkaloid isolated from the Red Sea sponges *Pseudoceratina arabica,* showed potent activity against the migration (in a wound-healing assay) and invasion (in the Cultrex^®^ BME cell invasion assay) of MDA-MB-231 (a highly metastatic phenotype) human breast cancer cells at nanomolar concentrations [203]. Cortistatin A, a steroidal alkaloid isolated from the marine sponge *Corticium simplex*, showed anti-angiogenic properties in a migration assay using a chemotactic chamber and in the Matrigel tubular formation assay. Cortistatin A also inhibited the migration and tubular formation of HUVECs induced by VEGF or bFGF at 2 nM [204]. Aeroplysinin-1 is a brominated alkaloid from the marine sponge *Aplysina aerophoba*. This compound inhibited the growth of endothelial cells in culture through an apoptotic process. It was also able to cause a decrease in the expression of matrix MMP-2 and urokinase and exhibited a dose-dependent inhibitory effect in the in vivo chorioallantoic membrane assay (CAM), showing potent apoptosis-inducing activity in the developing endothelium [205].

Motuporamines A, B and C, which are macrocyclic alkaloids containing a spermidine-like substructure isolated from *Xestospongia exigua* extracts, inhibited the in vitro invasion of basement membranes by MDA-231 breast carcinoma and PC-3 prostate carcinoma cells. Motuporamine C was the most potent inhibitor of cell migration and angiogenesis. This compound also changed the cytoskeletal structure, decreased the activation of β1-integrin (responsible for the adhesion and invasion of cancer cells) and inhibited cell migration and angiogenesis [206]. Bastadin 6, a macrocyclic tetramer alkaloid comprising brominated tyrosine derivatives that was isolated from the marine sponge *Lanthella* sp., showed an antiangiogenic effect through the inhibition of the vascular endothelial growth factor (VEGF)- or basic fibroblast growth factor (bFGF)-dependent proliferation of human umbilical vein endothelial cells (HUVECs), with 20-to 100-fold selectivity over normal fibroblasts. Another study showed that bastadins 9 and 16 (also isolated from the same sponge) together with bastadin 6 were able to display in vitro cytostatic, anti-angiogenic and antimigratory effects on mouse B16F10 melanoma cells [207]. It is proposed that these compounds exhibit selective induction activity against endothelial cells. Phidianidine A is an indole alkaloid isolated from the marine opisthobranch mollusk *Phidiana militaris* that significantly reduced CXCL12-induced migration in a rat pituitary adenoma cell line at 50 μM [208]. Sceptrin, a natural bromopyrrole imidazole alkaloid produced by various marine sponges, inhibited cell motility in several cancer cell lines by reducing cell contractility and binding to monomeric actin [209].

Latrunculins A and B are marine-derived macrolides isolated from the Red Sea sponge *Negombata magnifica*. These compounds are associated with disrupting the organization of cell microfilaments [210] and have antimigratory activity against highly metastatic human prostate cancer PC-3M-CT^+^ cells and murine brain-metastatic melanoma B16B15b cells [211,212]. Laulimalide is another macrolide (polyketide) isolated from marine sponge *Cacospongia mycofijiensis* that has the ability to inhibit cell proliferation with anti-tubulin activity (see Section 4.1). In addition, this compound exhibited cytotoxic activity at low doses and prevented blood vessel formation and VEGF-induced endothelial cell migration [213].

Among the terpenoids, the abovementioned triterpene glucoside ds-echinoside A (see Section 4.2), in addition to its effects on apoptosis regulation, inhibited the proliferation of human hepatocellular liver carcinoma cells (HepG2) with a IC_50_ value of 2.65 μM, suppressing cell adhesion, migration and invasion in a dose-dependent manner. The putative mechanism involved a decrease in NF-κB, VEGF and MMP-9 expression and an increase in tissue inhibitor of MMP-1 (TIMP-1) expression. Furthermore, this compound was able to reduce the tube formation of human endothelial cells and restrain neovascularization in the CAM assay [214]. Two sulfated triterpene glycosides (holothurin A and 24-dehydroechinoside A) isolated from *P. graeffei* significantly suppressed adhesion, invasion and migration in a HepG2 cellular model in a dose-dependent manner [215]. Another triterpene glucoside isolated from the marine cucumber *Cucumaria frondosa*, frondoside A, showed inhibition of migration and invasion at 0.1 to 1 μM in the human estrogen receptor-negative breast cancer cell line MDA-MB-231 in a wound-healing model assay and in the Matrigel invasion assay [178]. In this assay, the effect of frondoside A was higher on MDA-MB-231 breast cancer cell than on the non-tumorigenic MCF 10-A cell line derived from normal human mammary epithelium. In addition, in a in vivo assay, frondoside A (100 μg/kg/day i.p. for 24 days) strongly decreased the growth of MDA-MB-231 tumor xenografts in athymic mice without any manifest toxic side effects [216].

The sipholanes are triterpenes with a perhydrobenzoxepine (rings A and B) and bicyclodecane (rings C and D) conjugated ring system isolated from Red Sea sponge *Callyspongia siphonella* [217]. Three compounds of this family, sipholenol A, sipholenol E and sipholenone A, exhibited potent antimigratory activity in highly metastatic MDA-MB-231 breast cancer cells in a wound-healing assay [217]. Globostellatic acid X methyl esters (1–4) are triterpenes with an isomarabarican-type triterpenoidal skeleton isolated from the marine sponge *Rhabdastrella globostellata*. These compounds displayed potent inhibition of migration and tubular formation in human umbilical vein endothelial cells (HUVECs) induced by VEGF or bFGF [218].

Heteronemin, a natural sesterterpene isolated from the sponge *Heteronema erecta* [219], prevented tumor cell intravasation through the lymph-endothelial barrier in a three-dimensional (3D) cell culture model consisting of spheroids of the MCF-7 breast cancer cell [220].

The diterpenes pachycladins A and D, which were isolated from the Red Sea soft coral *Cladiella pachyclados* exhibited potent antimigratory activity when tested against prostate cancer PC-3 cells in a classical wound-healing assay. Sarcophines are marine-derived cembranoids (a class of diterpenes) with a 14-membered macrocyclic ring isolated from the soft coral *Sarcophytum glaucum* in the 1970s by Kashman and coworkers [221]. These complex compounds are described as one of the repellents that protect the coral against predators. Sarcophine exhibited potent anti-migration activity against highly metastatic mouse melanoma B16B15b cells [222] at micromolar concentrations. Similar results were obtained when using a semisynthetic analogue of sarcophine against MDA-MB-231 breast cancer cells and PC-3 prostate cells in wound-healing assays [223]. The diterpenes sinulodurins A and B isolated from the Palau soft coral *Sinularia dura* inhibited the invasion of highly metastatic prostate cancer PC-3M-CT^+^ cells in a 3D-spheroid disaggregation assay model [224]. Strongylophorine-26 is a meroditerpenoid isolated from the marine sponge *Petrosia* (*Strongylophora*) *corticata* [225] that inhibits the invasion of TNBC MDA-MB-231 cells and has antimigratory activity, as determined in an in vitro wound-healing assay.

Smenospongine, a sesquiterpene aminoquinone isolated from the marine sponge *Dactylospongia elegans*, displayed potent inhibition of the proliferation, migration and tube formation of human endothelial cells, suggesting favorable anti-angiogenic activity [226].

WA-25 is a synthetic dihydroaustrasulfone alcohol compound with anti-inflammatory activity derived from the marine compound WE-2 (austrasulfone), which was isolated from the soft coral *Cladiella australis* [227]. WA-25 showed activity by suppressing microvessel sprouting in organotypic rat aortic rings, and in endothelial cells, this compound was able, in a dose-dependent manner, to inhibit MMP-2/MMP-9 expression, proliferation, migration and tube formation in HUVECs.

Finally, although they do not fit in any of the previously mentioned cancer hallmarks, callyspongidiol and 14,15-dihydrosiphonodiol, which are polyacetylenediols isolated from marine sponges in the genus *Callyspongia* sp., are pharmacologically active substances that modulate immunity by the activation of human dendritic cells. These compounds might have effects on tumors or on autoimmune diseases [228].

### 4.4. Inhibitors of MAPKs

**Figure 4:**
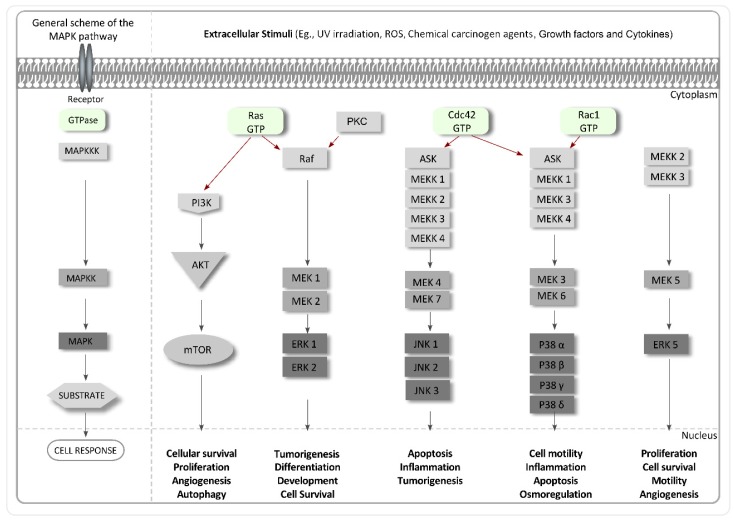
The relation between mammalian MAPK cascades and other kinases capable to induce cell responses involved in cancer. In MAPKs controlled signal transduction pathways after an extracellular stimuli transduced by a membrane receptor, the signal is conducted downstream by two kinases (MAPKKK and MAPKK) that finally arrives to a MAPK resulting in very functionally distinct responses (left part of the image). ERK is related to cellular survival, proliferation, angiogenesis and autophagy. JNK and p38 pathways are activated by MAPKKK (ASK) modulating tumorigenesis, cell motility, osmoregulation, inflammation and apoptosis. ERK 5 is a MAPK, which induces proliferation, cell survival, motility and angiogenesis. Finally, PI3K/AKT/mTOR axis is a serine-threonine protein kinases pathway induced by the GTPase Ras that plays an important role in cellular quiescence, proliferation and longevity and is an important regulator of oncogenesis and apoptosis in various types of cancers.

Mitogen-activated protein kinases (MAPKs) are a protein family that specifically phosphorylate the amino acids serine, threonine and tyrosine and are closely related to many cellular functions in cancer such as cell differentiation, proliferation and growth, cycle, apoptosis, migration, invasion and metastasis, energy metabolism and angiogenesis [229]. This family is activated by specific external factors and promotes the transduction of intracellular signals through the phosphorylation and activation of targets, which finally reach the nucleus and generate a cell response [230] (Figure 4). In many cases, MAPKs are considered as cellular amplifiers of biological signals, translating graded inputs into on or off outputs and filtering out low level noise [231]. MAPKs are frequently overexpressed or upregulated in cancer cells.

The most well-known MAPKs in mammals are MAPK/ERK kinase (MEK/ERK), protein kinase C (PKC), phosphoinositide 3-kinase (PI3K)/AKT, c-Jun N-terminal kinase (JNK), and p38 [232] (Figure 4). Cell proliferation and differentiation depend on epidermal growth factor (EGF), which induces the activation of receptor tyrosine kinase activity, of autophosphorylation and of the Ras/Raf/MEK/ERK signaling cascade [233]. Protein kinase C (PKC) is a family of phospholipid-dependent serine/threonine kinases involved in protein synthesis, gene expression, and cell proliferation, differentiation, and tumorigenesis. A wide range of distinct PKC enzymes have been found to be overexpressed in various cancers [234]. Phosphoinositide-3-kinase (PI3K)/AKT is a mitogen-activated protein kinase and a serine-threonine-specific protein kinase that plays an important role in cellular survival signaling and is an important regulator of oncogenesis and apoptosis in various types of cancers (Figure 4). This pathway is activated by membrane receptors such as VEGFR and PDGFR and is considered an important target in cancer research [235]. c-Jun N-terminal kinase (JNK) is another member of the MAPK family that regulates a range of biological processes, such as tumorigenesis and neurodegenerative disorders (Figure 4). This family is sensitive to stress stimuli, including cytokines, UV and γ-irradiation, cytotoxic drugs, DNA-damaging agents, and reactive oxygen species [236]. The p38 mitogen-activated protein kinase (MAPK) plays a key role in cellular adaptation to external stimuli such as stress response and inflammation and mediates cellular responses to UV irradiation, osmotic shock, ischemia, and DNA damage [237] (Figure 4). Finally, ERK5 is the most recently identified member of the mitogen-activated protein kinase (MAPK) family and is phosphorylated and activated by MEK5 (Figure 4). This kinase plays a critical role in cardiovascular development and vascular integrity and also in pathological conditions such as cancer and tumour angiogenesis.

Misregulated kinases are strongly correlated with oncological diseases [238]. As a result, many efforts have been made to develop kinase inhibitors as anticancer treatments (some of them are reported to be in either phase I or phase II clinical trials [239]). Marine sponges are an excellent source of protein kinase inhibitors, with over 70 novel compounds isolated to date [240]. The most novel and relevant of these are detailed in this section.

Hymenialdisine and debromohymenialdisine from the marine sponge *Stylotella aurantium* showed significant inhibitory activity against MEK. They affected the Raf/MEK/ERK cascade through the inhibition of the phosphorylation of ERK by MEK-1 [241]. Segraves et al. found that a methanol fraction of the sponge *Batzella* sp. exhibited inhibition of Raf kinase [242].

11-hydroxystaurosporine from the marine tunicate *Eudistoma* sp. [17] and bryostatin-1 from the marine bryozoan *Bugula neritina* [240] showed notable activity against PKC [243,244]. Lasonolide A (isolated from the Caribbean sponge *Forcepia* sp.) [245], penazetidine A (isolated from the marine sponge *Penares sollasi*) [246] and corallidictyals A and B (isolated from the marine sponge *Aka coralliphaga*) also showed potent inhibitory activity against PKC.

Andersen et al., in 2006, reported the isolation of the previously unknown meroterpenoid liphagal 1 via extraction from the methanol fraction of the sponge *Aka coralliphaga*, which is found in Dominica [247]. Liphagal 1 has a novel structure consisting of a new ‘liphagane’ type of meroterpenoid carbon skeleton. Recently, the total chemical synthesis of liphagal and of other analogues [248] has been reported. Liphagal 1 has shown a cytotoxic effect on LoVo (Human colon cancer cells) and Caco (Human colon cancer cells) cells with an IC_50_ below 0.7 µM and in MDA-468 (Human Breast cancer cells) with an IC_50_ of approximately 1.6 µM [249]. This compound also presented inhibitory activity against PI3K [249].

Dolastatin (previously mentioned in Section 4.1), along with the cyclooxygenase-2 inhibitor celecoxib, was able to inhibit the development of colon cancer in a rat model by increasing apoptotic occurrence and rate. Molecular docking analysis showed that both compounds had the capacity to interact with the active sites of PI3-K/AKT. Additionally, these drugs inhibited the increased expression of PI3-K and AKT [250]. Kahalalide F (a cyclic depsipeptide), which was isolated from the marine mollusk *Elysia rufescens*, inhibited the PI3K–AKT signaling pathway in the breast cancer cell lines SKBR3 and BT474 [251]. Elisidepsin trifluoroacetate (PM02734, Irvalec), a marine-derived cyclic peptide that belongs to the previously mentioned Kahalalide family of compounds (see Section 3), showed cytotoxic activity, causing cell death through the inhibition of the AKT/mTOR pathway [252].

Aplidine (dehydrodidemnin B, DDB, Aplidin), a cyclic depsipeptide isolated from the Mediterranean tunicate *Aplidium albicans,* had an antiproliferative effect on breast, melanoma and non-small-cell lung cancers. Its activity involved increasing oxidative stress, thus causing the activation of JNK and p38 MAPK phosphorylation and inducing apoptosis [253]. The heterocyclic alkaloids onnamide A and theopederin B, which were isolated from the marine sponge *Theonella* sp., inhibited protein synthesis and induced stress-activated protein kinases p38 and JNK activation in a mink lung epithelial cell line [254].

## 5. New Perspectives on the Virtual Screening of MNPs for the Discovery of Anticancer Compounds

Different people who develop cancer with identical histopathological diagnoses and clinical stages may exhibit highly diverse epigenetic and genetic characteristics, and this may be the cause of their different response to the same therapy. We must also know the molecular taxonomy of different human cancers in order to implement personalized therapy. Genomics, proteomics and metabolomics are already contributing and will continue to contribute to the molecular classification of cancer. This classification will potentially facilitate the development of new biomarkers that may become cancer therapeutic targets [255]. High-throughput DNA sequencing techniques are enabling us to detect large numbers of gene mutations in different types of cancer. However, most of these mutations are biologically irrelevant and therefore have no clinical significance. Only certain mutations confer on cancerous cells aberrant growth, survival, and drug resistance; these are the drive mutations. Accordingly, the proteins resulting from the expression of these mutated genes will be the specific targets of “smart drugs” for cancer [255]. Where should we look for new compounds capable of modulating the biological activity of oncogenic biomarkers with these drive mutations? Let us look at nature, which has been the pharmacy from which man has obtained medicines since antiquity. In this regard, marine natural products represent an important potential source of molecules with antineoplastic activity that must be explored. It is not economically feasible to test libraries of millions of compounds while looking for bioactive molecules. For this reason, it is necessary to implement a guided search via computational methods that reduces the vast chemical space to an experimentally approachable number of compounds. Molecular docking techniques are widely used for the study of protein-ligand interactions. After performing molecular docking experiments using AutoDock/vina software, the Gibbs free energy variation (ΔG, Kcal/mol) of a maximum of 20 poses/conformers is obtained for each ligand assayed. Compounds with lower calculated free energy variations (≤−10 kcal/mol) are selected as putative biomarker modulators [256,257]. *In silico* prediction of the ADMET (absorption, distribution, metabolism, excretion, and toxicity) properties of a compound provides us with additional filters for our selection of antitumor drug candidate compounds.

**Figure 5:**
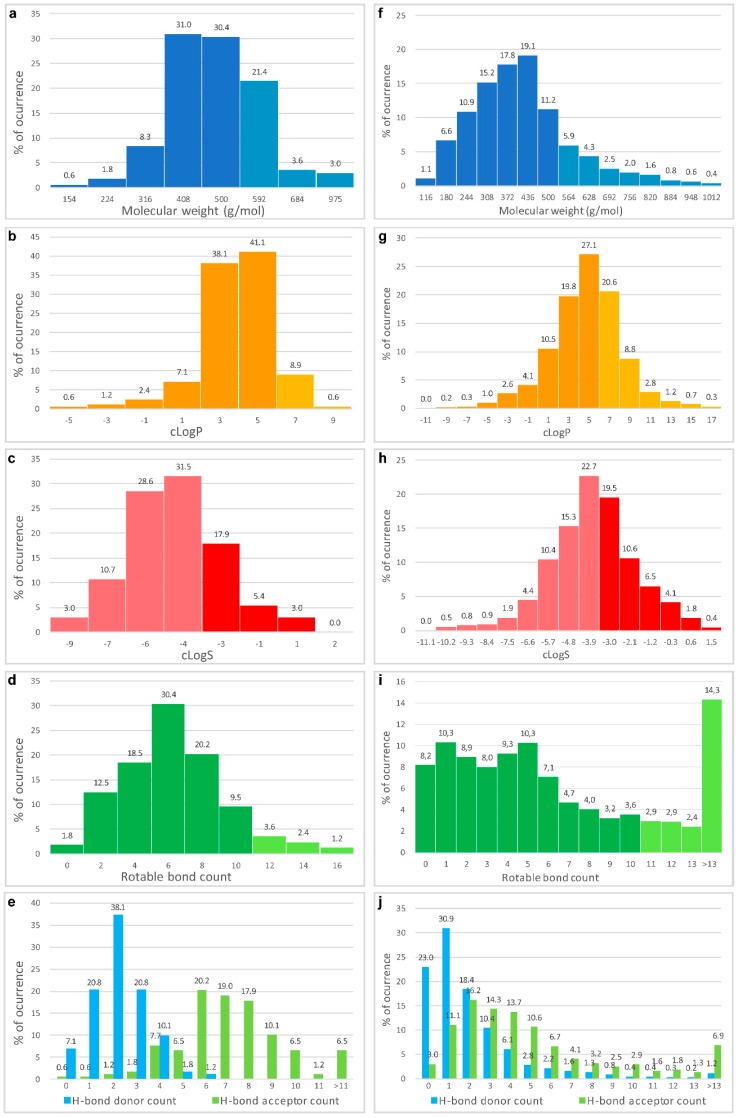
Distribution of the molecular weight (**a**,**f**), cLogP (**b**,**g**), cLogS (**c**,**h**), number of rotatable bonds (**a**,**f**) and number of hydrogen bond donors and acceptors (**d**,**i**) of the clinical oncologic drugs (**a**–**e**) and in the MNP database (**f**–**j**).

Traditionally, most antineoplastic drugs are administered parenterally, and although the oral route is gaining interest since it is safer and better tolerated by the patient, the parenteral route possess some advantages: it avoids the discomfort of injections and can even be done by patients at home. The physicochemical properties of these drugs and the physiological barriers make oral administration a difficult challenge to overcome [258]. The “Genomics of Drug Sensitivity in Cancer” project characterized more than 1000 cell lines and 168 anticancer agents, and their data are available at http://www.cancerrxgene.org/. We have calculated the ADMET profile of these anticancer compounds and provided unrestricted access to them through the website [259]. Each record in our database includes up to 47 ADMET calculated parameters, and the molecular structure in MOL2 format is available for molecular docking purposes. A partial analysis of these data is shown in Figure 5. Interestingly, these data clearly demonstrate the above assertion that many anticancer agents have poor physicochemical properties. For example, Figure 5a shows that 28% of these compounds have a molecular weight greater than 500; additionally, approximately 10% have a cLogP greater than 5.0 (Figure 5b), and only 26% have a cLogS greater than 4 (Figure 5c).

As an additional effort to improve the contribution of this paper, we have built a database of marine natural compounds by using data mining techniques to find the structural formulas of these compounds. The structures in MOL2 format and the ADMET profile of all compounds have been included in the database, which is available at the following address: [260]. It was reported that approximately 1% of the marine samples tested had potential antitumor activity compared to 0.1% of the terrestrial samples tested [261]. The systematic study of MNPs began almost 70 years ago with the pioneering works of Werner Bergmann and was continued by John Blunt and Murray Munro at the University of Canterbury, New Zealand, who created the MarinLit database [262]. From this work, we have extracted the CAS number of 20,451 molecules, of which 17,145 have been identified in the SciFinder database. To convert CAS numbers to chemical structures in an electronic format, we have used the NIH Chemical Identifier Resolution Service available at [263]. Using this service, we have managed to resolve 14,493 compounds with a molecular weight of less than 7000. These compounds are the ones that are currently included the database of marine compounds fully accessible at the aforementioned address.

The right panels (f–j) in Figure 5 show analysis of selected physicochemical characteristics of the 14,442 marine compounds in our database, thus allowing us to compare them with the data shown in the left panels (a–e), which refer to the 168 compounds with anticancer activities tested by the “Genomics of Drug Sensitivity in Cancer” project. A few interesting observations should be made when the two databases, i.e., our own MNPs database and the anticancer compounds database, are compared. The frequency diagram shown in Figure 5f indicates that 82% of the MNPs in our database have a molecular weight lower than or equal to 500, and 99.6% of all compounds have a value similar to the maximum of the anticancer compounds (see Figure 5a); of the latter, 28% have an MW greater than 500. If we analyze the cLogP values of MNPs, we can observe that 95% of the compounds have a cLogP equal to or less than 9 (Figure 5g), which is the highest value found in the anticancer compounds (Figure 5a). Considering the cLogS value, we can see that 93% of MNPs (Figure 5h) are within the range of the anticancer compounds (Figure 5c).

The oral bioavailability of drug candidates can be correlated with molecular flexibility, as measured by the number of rotatable bonds, polar surface area and total hydrogen bond count, which is independent of molecular weight [264]. Compounds with 10 or less rotatable bonds, a polar surface area of less than 140 Å2 and 12 or fewer H-bond donors and acceptors have a high probability of exhibiting good oral bioavailability. When we compare the data of the number of rotatable bonds of the marine compounds (Figure 5i) with that of the anticancer compounds (Figure 5d), we can observe that 77% and 93%, respectively, satisfy that condition. The comparison of the polar surface area values (data not shown) in both types of compounds yields similar values for the two groups, specifically, 84% (marine compounds) and 87% (anticancer compounds). Finally, the analysis of H-bond donors (HBDs) and acceptors for both sets of compounds also affords similar values: 86% (marine compounds) and 83% (anticancer compounds) have 5 or fewer HBDs and 10 or fewer H-bond acceptors. Taken together, these data show that most of the MNPs collected in our database have a high potential as lead compound candidates. Research focused on testing this library of compounds *in silico* against different targets of interest in cancer, in conjunction with in vitro verification in different cancer cell lines, is ongoing. Similarly, a recent paper on a database of natural compounds that have been tested in cancer cell lines will become a valuable tool to guide our search for target proteins in our *in silico* experiments [265]. We have analyzed the data presented in that database and showed a total of 127 proteins (data not shown), along with their UNIPROT code, that are targets for different compounds. In fact, we have abundant high-resolution structural information in the Protein Data Bank for many of these proteins, and therefore, it is very likely that the molecular docking data will soon provide us with several marine compounds that are good candidates for inhibiting some of these protein cancer biomarkers.

## 6. Limitations of Marine Invertebrates as Source for Anticancer Agents

Given the above, it has now been well established that the use of bioactive compounds of marine origin will bring increasing attraction in the following years as an alternative for the discovery of new drugs. Nevertheless, some limitations of the search for marine compounds from invertebrates must be considered, including the low amounts in which these products are produced by the organisms, the potential presence of toxins and inorganic salts derived from the organisms or environment, the wide diversity of chemical compounds produced by an organism, and the existence of nonspecific pharmacological targets.

To develop in vitro screening assays, small amounts are needed; however, in preclinical studies, hundreds of grams to kilograms are often required for testing purposes. Currently, this obstacle is overcome using a combination of novel techniques in chemical synthesis and improving harvest, aquaculture or isolation processes [266]. Another limiting factor in the use marine organisms is the potential presence of toxins from the organisms or of environmental origin and the presence of inorganic salts. These species may compromise the use of raw extracts for in vitro screening purposes. Often, the production of toxins by a marine invertebrate is an indicator that this organism is a candidate source of bioactive compounds. Therefore, an effort should be made to characterize the possible contaminants (inorganic salts and toxins) in order to make marine extracts compatible with in vitro testing. Many analytical techniques are currently available for the analysis, isolation, characterization and separation of active compounds in marine extracts [267,268]. The dependency of the variety of chemical compounds (chemotype) produced by an organism on environmental conditions might be solved using controlled aquaculture techniques. Controlled aquaculture not only could avoid the problem of exhausting the marine resources but also could be a feasible option to produce the required biomass for the high scale production needed in a drug discovery pipeline. Moreover, improvements in chemical synthesis techniques and combinatorial chemistry are providing satisfactory solutions for appropriate sourcing [269,270]. Finally, the molecular targets for most of the newly discovered MNPs are unknown; therefore, high-throughput screening techniques, together with Omics and virtual screening, should be integrated to overcome the limitations of in vitro techniques.

## 7. Conclusions

This review summarizes the most recently isolated, derived or synthetically obtained compounds from marine invertebrates that have exhibited potential as cancer therapies within the last two decades. Undoubtedly, marine resources provide priceless and unexploited biochemical diversity and show a greater potential than plant resources for the discovery of new anticancer drugs, with approximately 18% of all MNPs discovered so far. The increasing number of publications on MNPs in the last few years reflects this fact.

A significant number of marine compounds have shown potential anticancer activities against the different hallmarks of cancer, such as cell growth inhibition, antimitotic activity (anti-tubulin effects), apoptosis and/or autophagy induction, and migration, invasion or metastasis inhibition. For most of the isolated compounds and their semisynthetic derivatives, the reported activities have been observed in vitro, but a significant number of these compounds, i.e., more than twenty, are either approved or in clinical phase. MNPs exhibit extensive chemical variability and complexity and include alkaloids, polyketides, terpenes, peptides and carbohydrates. Although marine resources are also limited, sourcing issues should be solved in the future by using aquaculture and biotechnology processes and the improvement of chemical synthesis in order to provide sufficient amounts of compounds for research purposes or pharmaceutical development.

Classical approaches based on the screening of extracts derived from marine organisms using in vitro techniques have limited applicability. The use of Omics has been extended to research on MNPs for anticancer drug discovery, offering new opportunities for the discovery of more specific cancer therapeutic targets or biomarkers. This will certainly enable the development of “precision medicine” in the near future. This large in vitro screening is generating a limitless number of compounds that require the use of virtual screening or computational methods to reduce the vast chemical space (thousands of compounds) to an experimentally approachable number of compounds. The combination of Omics techniques with virtual screening for the selection of compounds against particular cancer targets is becoming the method of choice in this area. Nevertheless, caution should be exercised in the selection of anticancer compounds for virtual screening. The comparison performed in this review, which includes the properties of the 168 most promising anticancer compounds from the GDSC database, demonstrates that using regular filtering techniques based on ADMET parameters may rule out many compounds with potential anticancer activity. Finally, a new public access MNPs library is reported for *in silico* purposes and is already being tested for different targets of interest in cancer [260].

## Figures and Tables

**Table 1:** List of FDA- and EMEA-approved marine anticancer drugs from an invertebrate source.

Organization and Year	Compound Name	Marine Organism	Chemical Class	Disease Area	Mode of Action	Company or Institution	Refs.
FDA 1969	Cytarabine (Ara-C)	Sponge	Nucleoside	Anticancer	DNA polymerase inhibitor	Bedford, Enzon	[37]
FDA 2004	Ziconotide	Cone snail	Peptide	Pain	Modulator of neuronal calcium channels	Neurex Corp	[38]
EMEA 2007	Trabectedin (E7389)	Tunicate	Alkaloid	Anticancer	Inhibits cancer cell growth of and affects the tumor microenvironment	PharmaMar	[39]
FDA 2010	Eribulin mesylate (E7389)	Sponge	Macrolide	Anti-breast cancer	Microtubule interfering agent	Eisai Inc.	[40]
FDA 2011	Brentuximab vedotin (SGN-35)	Mollusk	Antibody-drug conjugate	Lymphoma	CD30-directed antibody-cytotoxic drug conjugate	Seattle Genetics Inc.	[41]

**Table 2:** List of marine drugs in clinical trials.

Clinical Status	Compound Name	Marine Organism	Chemical Class	Disease Area	Mode of Action	Company or Institution	Refs.
Phase III	Plitidepsin	Tunicate	Depsipetide	Anti-cancer	Induces cell cycle arrest or apoptosis	PharmaMar	[76]
	Gemcitabine (GEM) (Gemzar)	Sponge	Nucleoside	Anti-cancer	Ribonucleotide reductase inhibitor Replaces cytidine during DNA replication	Eli Lilly and Company	[77]
Phase II	Glembatumumab vedotin	Mollusk	Antibody drug conjugate	Breast cancer and melanoma	Targets glycoprotein NMB (a protein overexpressed by multiple tumor types)	Celldex Therapeutics	[78]
	Elisidepsin	Mollusk	Depsipetide	Anti-cancer	Antineoplastic agent, modifiying lipids from cell membrane	PharmaMar	[79]
	PM1004	Nudibranch	Alkaloid	Anti-cancer	DNA-binding	PharmaMar	[80]
	Pseudopterosins	Soft coral	Diterpen glycoside	Wound healing	Eicosanoid metabolism	The Regents Of The University Of California	[81]
	IPL576,092 (Contignasterol derivative)	Sponge	Miscellaneous	Anti-inflammatory	Inhibition of leucocyte infiltration and hypersensitivity during allergy	Aventis Pharma	[82]
Phase I/II	PM-10450 (Zalypsis^®^)	Sponge	Alkaloid	Anti-cancer drug	Transcription inhibitor	PharmaMar	[83]
	Discodermolide	Sponge	Polyketide	Anti-cancer drug	Microtubule interfering agent	Novartis	[84]
Phase I	Bryostatin-1	Bryozoa	Polyketide	Anti-cancer drug	Protein kinase C	National Cancer Institute	[85]
	Pinatuzumab vedotin	Mollusk	Antibody drug conjugate	Non-Hodgkin lymphoma, leukemia	Apoptosis stimulant; Mitosis inhibitor and Tubulin inhibitor	Genentech, Inc.	[86]
	Tisotumab Vedotin (HuMax^®^-TF-ADC)	Mollusk	Antibody drug conjugate	Ovarian, endometrium, cervix and prostate cancer	Antineoplastic, Drug conjugate, Immunotoxin and monoclonal antibodies	Genmab and Seattle Genetics	[87]
	HT1286 (Hemiasterlin derivative)	Sponge	Tripeptide	Anti-cancer drug	Microtubule interfering agent	Wyeth	[84]
	LAF389 (Bengamide B derivative)	Sponge	Peptide	Anti-cancer drug	Methionine aminopeptidase inhibitor	Novartis	[84]
	Hemiasterlin (E7974)	Sponge	Tripeptide	Anti-cancer drug	Microtubule interfering agent	Eisai Inc.	[84]
	PM-060184	Sponge	Polyketide	Anti-cancer drug	Microtubule interfering agent	PharmaMar	[88]
	NVP-LAQ824 (Psammaplin derivative, Dacinostat)	Sponge	Miscellaneous	Anti-cancer drug	Histone deacetylase (HDAC) inhibitors or DNA methyltransferases (DNMT) inhibitor	Novartis Pharma	[89]

## Supplementary Materials

Supplementary File 1

## Abbreviations

ADMET	absorption, distribution, metabolism, excretion, and toxicity
AMPK	AMP-activated protein kinase
CAM	chorioallantoic membrane assay
MNPs	marine natural products
MAPK	mitogen-activated protein kinase
VEGF	vascular endothelial growth factor
ERS	endoplasmic reticulum stress
MMP	matrix metalloproteinase
